# Predictors and Risk Factors of Pathologic Complete Response Following Neoadjuvant Chemoradiotherapy for Rectal Cancer: A Population-Based Analysis

**DOI:** 10.3389/fonc.2019.00497

**Published:** 2019-06-13

**Authors:** Yinuo Tan, Dongliang Fu, Dan Li, Xiangxing Kong, Kai Jiang, Liubo Chen, Ying Yuan, Kefeng Ding

**Affiliations:** ^1^Department of Medical Oncology, The Second Affiliated Hospital of Zhejiang University School of Medicine, Hangzhou, China; ^2^Key Laboratory of Cancer Prevention and Intervention, China National Ministry of Education, Key Laboratory of Molecular Biology in Medical Sciences, Cancer Institute, The Second Affiliated Hospital of Zhejiang University School of Medicine, Hangzhou, China; ^3^Department of Colorectal Surgery, The Second Affiliated Hospital of Zhejiang University School of Medicine, Hangzhou, China

**Keywords:** rectal cancer, pathologic complete response, neoadjuvant chemoradiotherapy, mucinous adenocarcinoma, SEER

## Abstract

**Background:** Patients with rectal cancer who achieve pathologic complete response (pCR) after neoadjuvant chemoradiotherapy (nCRT) may have a better prognosis and may be eligible for non-operative management. The aim of this research was to identify variables for predicting pCR in rectal cancer patients after nCRT and to define clinical risk factors for poor outcome after pCR to nCRT and radical resection in rectal cancer patients.

**Methods:** A retrospective review was performed using the Surveillance, Epidemiology, and End Results (SEER) database from 2004 to 2013. Non-metastatic rectal cancer patients who received radical resection after neoadjuvant chemoradiotherapy were included in this study. Multivariate analysis of the association between clinicopathological characteristics and pCR was performed, and a logistic regression model was used to identify independent predictors for pCR. A nomogram based on the multivariate logistics regression was built with decision curve analyses to evaluate the clinical usefulness.

**Results:** A total of 6,555 patients were included in this study. The proportion of patients with pCR was 20.5% (*n* = 1,342). The nomogram based on multivariate logistic regression analysis showed that clinical T4 and N2 stages were the most significant independent clinical predictors for not achieving pCR, followed by mucinous adenocarcinoma and positive pre-treatment serum CEA results. The 3-year overall survival rate was 92.4% for those with pCR and 88.2% for those without pCR. Among all the pCR patients, mucinous adenocarcinoma patients had the worst survival, with a 3-year overall survival rate of 67.5%, whereas patients with common adenocarcinoma had an overall survival rate of 93.8% (*P* < 0.001). Univariate and multivariate analyses showed that histology and clinical N2 stage were independent risk factors.

**Conclusion:** Mucinous adenocarcinoma, positive pre-treatment serum CEA results, and clinical T4 and N2 stages may impart difficulty for patients to achieve pCR. Mucinous adenocarcinoma and clinical N2 stage might be indicative of a prognostically unfavorable biological tumor profile with a greater propensity for local or distant recurrence and decreased survival.

## Introduction

Colorectal cancer is the third leading cause of cancer death worldwide (1, 2). Recent studies suggest that 1.2 million new patients are diagnosed annually worldwide, among whom approximately half a million would die of this disease (3). Neoadjuvant chemoradiotherapy (nCRT), followed by radical surgical resection, is the most recommended routine management for locally advanced rectal cancer (4–6). nCRT has been proven to downstage tumors and improve surgical outcomes, which would eventually turn out to be a long-term oncologic outcome (5, 6). Some rectal cancer patients who received nCRT achieve a pathologic complete response (pCR), which is always associated with a better outcome than that of patients who had residual tumor after nCRT. Total mesorectal excision (TME) is a standard surgery for rectal cancer (6), and it may lead to a risk of morbidity, including bowel, urinary and sexual function impairment, and a temporary or permanent ostomy (7). More and more research is focused on the “watchful waiting” approach as an alternative to radical resection, which means that those who achieved clinical complete response (cCR) following nCRT are monitored closely instead of receiving surgery (4, 8, 9). Patients with low-recurrence-risk tumors or those who achieve pCR may be more suitable for this strategy (8, 10). Thus, the ability to predict factors for pCR or factors associated with a high risk of recurrence is needed, which may help clinicians select patients who may be more suitable for the “watchful waiting” approach after nCRT. The aim of this research was to identify clinicopathological factors that predict pCR and overall outcome after nCRT and TME surgery.

## Patients and Methods

### Patient Selection

**Figure 1:**
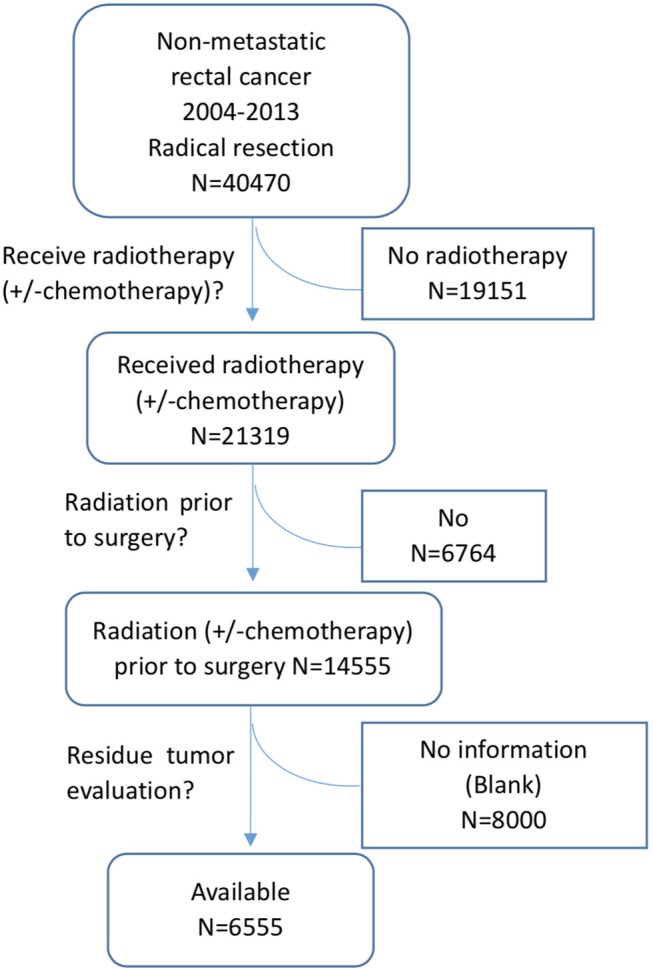
Filtering process of patient data from the SEER database.

The data in this study were extracted from the SEER database as previously reported (11, 12). The SEER database is a National Cancer Institute-based authoritative source of cancer data in the United States. It collects and publishes cancer incidence and survival data from 18 population-based cancer registries that cover ~28% of the US population (1). SEER^*^Stat is an online program provided by SEER to obtain patient information. This study was approved by the ethics committee of the Second Affiliated Hospital of Zhejiang University College of Medicine. Patients were eligible for inclusion if they had histologically confirmed, primary, non-metastatic rectal adenocarcinoma and received curative resection after nCRT. Patients were excluded if they had a lifetime history of another primary malignancy, had *in situ* cancer, or died within 1 month after the operation. Patients without residual tumor information were also excluded. The detailed selection process is shown in Figure 1. As we don't know whether chemotherapy was administered together with radiation, all the patients received radiotherapy with or without chemotherapy were included in the selection process, noted as radiotherapy (± chemotherapy).

### Clinicopathological Data

The patient demographics (age, gender, race, and marital status), tumor characteristics (differentiation, clinical T and N stage, tumor histology, and pre-treatment CEA level) and survival data were acquired from the SEER database.

For the univariate and multivariate analyses, the variables were analyzed as discrete categorical variables. A median age of 50 was chosen as the cut-off value. Patients with a survival time of less than 1 month were excluded because such patients may have died of surgical complications.

### Statistical Analysis

Univariate and multivariate logistic regression models were used to identify factors predicting pCR. A nomogram was constructed based on statistically significant factors identified by the multivariate analysis from the logistic regression model to predict the possibility of pCR. Decision curve analysis (DCA) was used to assess the clinical usefulness and net benefits of the prediction model for achieving pCR. The primary endpoint of this study, colorecal cancer cancer-specific survival (CSS), was calculated from the date of diagnosis to the date of cause-specific death. Clinicopathological variables were tested for independence by Pearson χ^2^ tests. CSS was analyzed using Kaplan-Meier survival methods, and the 3-year CSS was estimated by Kaplan-Meier survival curves. Log-rank tests were employed to assess statistical significance. Multivariate analyses using Cox proportional hazards models were used to identify independent prognostic factors for cause-specific survival. Statistical analyses were performed and graphics were created using IBM SPSS Statistics software version 22.0 (IBM Corporation, Armonk, NY, USA). The nomogram analysis and DCA were conducted using R version 3.5.1 (https://www.r-project.org/). Statistical significance was set at a two-sided *P-*value < 0.05.

## Results

A total of 6,555 patients were included in our study. The proportion of patients with pCR was 20.5% (*n* = 1,342). The clinical and pathological features of all patients are shown in Table 1. Among the variables, no significant differences were found in age, gender, marital status or race between the pCR and non-pCR groups, but there were differences in histology (*P* < 0.001), pre-treatment serum CEA results (*P* = 0.006), clinical T stage (*P* < 0.001) and N stage (*P* < 0.001).

**Table 1:** Patient demographics and tumor characteristics.

**Variable**	**non-pCR (%)**	**pCR(%)**	***P*-value**
Age			0.197
≤ 50 years	1,201 (81%)	287 (19%)	
>50 years	4,012 (79%)	1,055 (21%)	
Gender			0.545
Male	1,973 (79%)	520 (21%)	
Female	3,240 (80%)	822 (20%)	
Marital status			0.094
Married	3,073 (79%)	804 (21%)	
Single	912 (82%)	206 (18%)	
Divorced	1,003 (80%)	258 (20%)	
Unknown	225 (75%)	74 (25%)	
Race			0.313
White	4,198 (79%)	1,087 (21%)	
Black	437 (82%)	95 (18%)	
Other	560 (79%)	153 (21%)	
Unknown	18 (72%)	7 (28%)	
Histology			<0.001
Common adenocarcinoma	4,846 (79%)	1,283 (21%)	
Mucinous adenocarcinoma	367 (86%)	59 (14%)	
Differentiation		<0.001
Well	327 (80%)	83 (20%)	
Moderate	3,753 (81%)	908 (19%)	
Poor	511 (81%)	121 (19%)	
Undifferentiated	67 (86%)	11 (14%)	
Unknown	555 (72%)	219 (28%)	
CEA			0.006
Negative	1,928 (78%)	543 (22%)	
Positive	1,576 (82%)	348 (18%)	
Unknown	1,709 (79%)	451 (21%)	
cT stage			<0.001
1	278 (73%)	104 (27%)	
2	652 (77%)	193 (23%)	
3	3,768 (79%)	975 (21%)	
4	515 (88%)	70 (12%)	
cN stage			
0	2,477 (77%)	729 (23%)	0.000
1	2,072 (80%)	513 (20%)	
2	664 (87%)	100 (13%)	
Total	5,213 (80%)	1,342 (20%)	

### Predictors of pCR

The proportion of patients who received pCR was ~20.47% (1,342/6,555). Patients with clinical T4 tumors (12%, 70/585) were significantly less likely to reach pCR (*P* < 0.001) than were those with lower T stage tumors (T1, 27%, 104/382; T2, 23%, 193/845; T3, 21%, 975/4,743). Patients with clinical N2 tumors (13%, 100/764) were significantly less likely to reach pCR (*P* < 0.001) than were those with lower N stage tumors (N0, 23%, 729/3,206; N1, 20%, 513/2,585).

Additionally, patients whose pathological diagnosis was common adenocarcinoma (21%, 1,283/6,192) were significantly more likely to achieve pCR (*P* < 0.001) than were those diagnosed with mucinous adenocarcinoma (14%, 59/426). Moreover, patients with positive pre-treatment serum CEA results (18%, 348/1,924) were significantly less likely to reach pCR (*P* = 0.006) than were negative pre-treatment serum CEA patients (22%, 543/2,471).

The details about the univariate analyses and multivariate analyses of predictors with logistic regression models for pCR are shown in Table 2. It appears to be significantly harder for patients who were diagnosed with mucinous adenocarcinoma to reach pCR [odds ratio (OR) = 0.61, (*P* = 0.001)]. The patients with positive pre-treatment serum CEA results (OR = 0.78, *P* = 0.001), clinical T4 stage (OR = 0.36, *P* < 0.001) and N2 stage (OR = 0.51, *P* < 0.001) were also included.

**Table 2:** Univariate analyses and multivariate analyses of predictors for pCR using logistic regression models.

**Variable**	**Univariate analyses**		**Multivariate analyses**	
	**Odds Ratio (95% CI)**	***P*-value**	**Odds ratio (95% CI)**	***P-*value**
**AGE**				
≤ 50 years	1		1	
>50 years	1.1 (0.95–1.27)	0.198	1.05 (0.91–1.22)	0.511
**GENDER**				
Male	1		1	
Female	0.96 (0.85–1.09)	0.545	0.96 (0.84–1.09)	0.500
**MARRIAGE STATUS**				
Married	1		1	
Single	0.86 (0.73–1.02)	0.090	0.93 (0.78–1.1)	0.402
Divorced	0.98 (0.84–1.15)	0.832	1.02 (0.87–1.2)	0.814
Unknown	1.26 (0.96–1.65)	0.102	1.22 (0.92–1.61)	0.161
**RACE**				
White	1		1	
Black	0.84 (0.67–1.06)	0.139	0.83 (0.66–1.06)	0.132
Other	1.06 (0.87–1.28)	0.581	1.06 (0.88–1.29)	0.532
Unknown	1.5 (0.63–3.6)	0.363	1.47 (0.6–3.56)	0.397
**HISTOLOGY**				
Common adenocarcinoma	1		1	
Mucinous adenocarcinoma	0.61 (0.46–0.8)	0.001	0.68 (0.51–0.9)	0.008
**DIFFERENTIATION**				
Well	1		1	
Moderate	0.95 (0.74–1.23)	0.709	0.96 (0.74–1.23)	0.733
Poor	0.93 (0.68–1.27)	0.663	1.08 (0.79–1.48)	0.626
Undifferentiated	0.65 (0.33–1.28)	0.21	0.81 (0.41–1.61)	0.544
Unknown	1.55 (1.17–2.07)	0.003	1.58 (1.18–2.11)	0.002
**CEA**				
Negative	1		1	
Positive	0.78 (0.67–0.91)	0.001	0.83 (0.71–0.97)	0.016
Unknown	0.94 (0.81–1.08)	0.365	0.92 (0.8–1.06)	0.244
**cT STAGE**				
1	1		1	
2	0.79 (0.6–1.04)	0.097	0.86 (0.65–1.13)	0.278
3	0.69 (0.55–0.88)	0.002	0.79 (0.62–1)	0.050
4	0.36 (0.26–0.51)	<0.001	0.44 (0.31–0.61)	<0.001
**cN STAGE**				
0	1		1	
1	0.84 (0.74–0.96)	0.008	0.88 (0.77–1)	0.052
2	0.51 (0.41–0.64)	<0.001	0.57 (0.45–0.72)	<0.001

**Figure 2:**
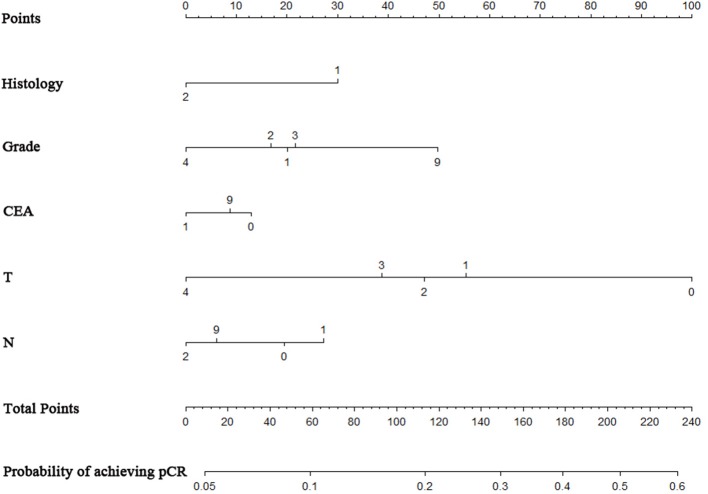
Nomogram to predict the probability of achieving pCR. The factors of grade, histology, CEA, T-classification, and N-classification were included in the model. Histology: “1” = adenocarcinoma, “2” = mucinous adenocarcinoma; grade: “1” = well, “2” = moderate, ”3” = poor, “4” = undifferentiated; T stage classification: “1” = T1, “2” = T2, “3” = T3, “4” = T4. N stage classification: “1” = N1, “2” = N2.

A nomogram was constructed based on statistically significant factors identified by multivariate logistic regression to predict the possibility of achieving pCR. T4 was the most predominant prognostic factor, followed by N2, poor differentiation grade, and positive pre-surgery CEA results (Figure 2). A vertical line was drawn from the factor to the point scale to determine the scores of all risk factors (detailed in Supplement Table 2). We then summarized all the discrete values and drew a straight line from the total scale to lines estimating the possibility of achieving pCR to obtain the individual's possibility.

**Figure 3:**
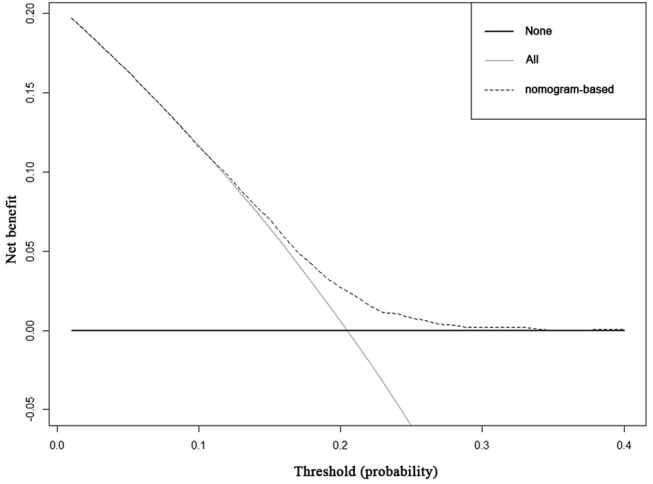
Decision curve analysis for the nomogram for predicting pCR. The x-axis is the risk threshold probability that changes from 0 to 1, and the y-axis is the calculated net benefit for a given threshold probability. The dashed lines depict the net benefit of the risk model–based selection strategy for screening, whereas the black and gray lines display the net benefits in the alternative strategies of screening all patients (black) vs. screening no patients (gray) in the data set.

Decision curve analysis to inform clinical decisions was better than a scenario in which all patients or no patients are treated across a wide range of thresholds between 0.10 and 0.34 (Figure 3). The calibration curve for predicting probability of achieving pCR was shown in Supplement Figure 1.

### Risk Factors Among pCR Patients

**Figure 4:**
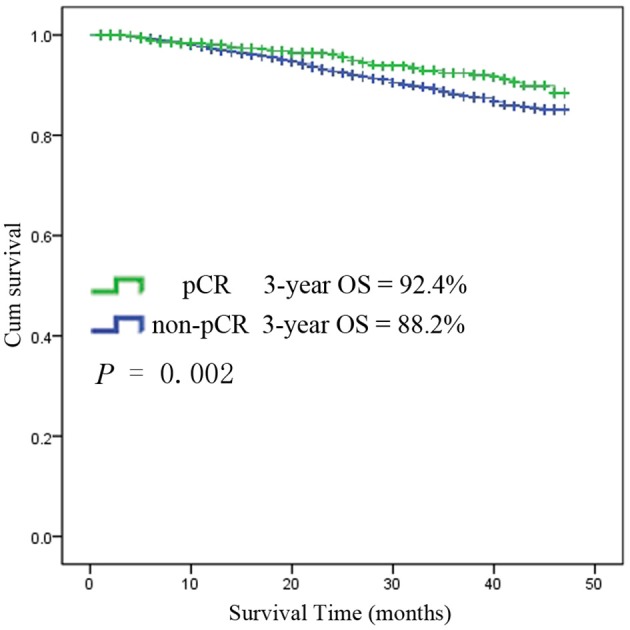
Kaplan-Meier survival curves for patients with and without pCR.

**Figure 5:**
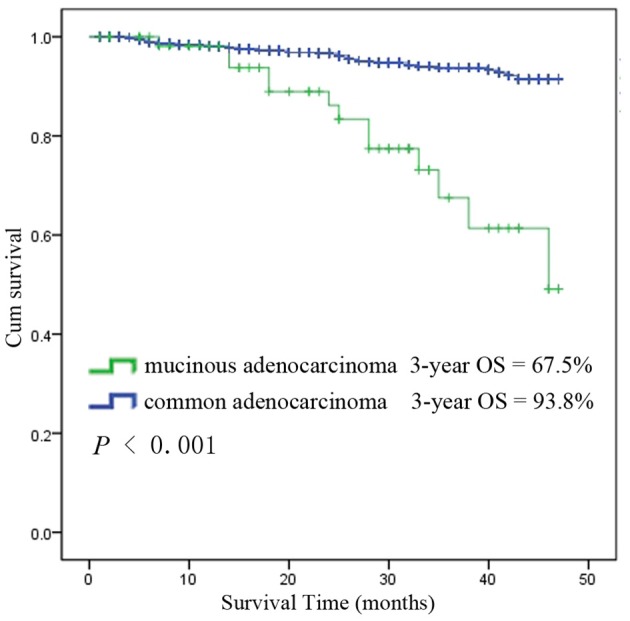
Kaplan-Meier survival curves for mucinous adenocarcinoma and common adenocarcinoma among all patients with pCR.

Among the 6,555 rectal cancer patients, the 3-year overall survival rate was 92.4% for those with pCR and 88.2% for those without pCR. The clinical and pathological characteristics of pCR patients was shown in Supplement Table 1. Among all the pCR patients, mucinous adenocarcinoma patients had the worst survival, with a 3-year overall survival rate of 67.5%, while those with common adenocarcinomas had a rate of 93.8% (*P* < 0.001). Kaplan-Meier survival curves for patients with and without pCR are shown in Figure 4. Kaplan-Meier survival curves for patients with mucinous adenocarcinoma and common adenocarcinoma are shown in Figure 5.

The results of the univariate and multivariate analysis using the Cox regression model among patients who achieved pCR are demonstrated in Table 3. The adjusted HR from the univariate analyses indicates that patients with mucinous adenocarcinoma had a significantly poorer survival rate than did those with common adenocarcinoma (HR 5.04, 95% CI 2.74–9.26; *P* < 0.001). Similarly, the adjusted HR for clinical N stage indicates that patients with N2 stage tumors had a worse prognosis than that of patients with lower N stage tumors (HR 4.19, 95% CI 2.17–8.1; *P* < 0.001).

**Table 3:** Univariate and multivariate analyses using Cox models among patients who achieved pCR.

**Variable**	**Univariate analysis**		**Multivariate analysis**	
	**Hazard ratio (95% CI)**	***P-*value**	**Hazard ratio (95% CI)**	***P-*value**
**AGE**				
≤ 50 years	1.00		1.00	
>50 years	1.93 (0.92–4.04)	0.082	1.04 (1.01–1.06)	0.001
**GENDER**				
Male	1.00		1.00	
Female	1.48 (0.87–2.53)	0.148	1.66 (0.94–2.94)	0.080
**RACE**				
White	1.00		1.00	
Black	0.58 (0.18–1.87)	0.365	0.71 (0.22–2.31)	0.572
Other	0.76 (0.33–1.77)	0.529	0.94 (0.4–2.21)	0.883
**MARRIAGE STATUS**				
Married	1.00		1.00	
Single	0.65 (0.27–1.54)	0.325	0.78 (0.32–1.89)	0.583
Divorced	1.19 (0.64–2.2)	0.583	1.35 (0.7–2.6)	0.369
**HISTOLOGY**				
Common adenocarcinoma	1.00		1.00	
Mucinous adenocarcinoma	5.04 (2.74–9.26)	<0.001	2.92 (1.48–5.74)	0.002
**DIFFERENTIATION**				
Well	1.00		1.00	
Moderate	0.79 (0.28–2.22)	0.651	0.77 (0.27–2.2)	0.628
Poor	3.08 (1.03–9.23)	0.044	1.9 (0.61–5.94)	0.268
Undifferentiated	4.82 (0.88–26.46)	0.070	3.79 (0.63–22.97)	0.147
**CEA**				
Negative	1.00		1.00	
Positive	1.15 (0.62–2.14)	0.663	1.13 (0.6–2.14)	0.712
**cT STAGE**				
1	1.00		1.00	
2	0.84 (0.24–2.98)	0.788	0.78 (0.21–2.81)	0.701
3	1.4 (0.51–3.89)	0.515	1 (0.35–2.87)	1.000
4	3.2 (0.94–10.95)	0.063	2.68 (0.74-9.76)	0.135
**cN STAGE**				
0	1.00		1.00	
1	1.26 (0.73–2.17)	0.407	1.16 (0.66–2.03)	0.602
2	4.19 (2.17–8.1)	<0.001	3.3 (1.61–6.75)	0.001

## Discussion and Conclusions

### Predictors of pCR

The rates of pCR after nCRT for rectal cancer vary from 10 to 25% or higher in the literature.(5, 13–29) In our study using the SEER database, the rate of non-metastatic rectal cancer patients who achieved pCR between 2004 and 2013 was 20.5%, ranging from 13 to 28% among different subgroups. Several clinicopathologic factors were identified in our study to be associated with pCR, including non-mucinous adenocarcinoma, negative pre-treatment serum CEA, and non-T4 clinical and non-N2 clinical stages. Numerous retrospective cohort studies have previously identified a variety of disease-related and treatment-related variables as potential predictors of pCR, including tumor differentiation, tumor size, pre-treatment CEA, clinical T and N stages, circumferential tumor extent, whether the CEA decrease reaches 75%, radiation dose, and the interval from the end of radiation to surgery (5, 10, 16, 18, 21–25, 28, 30–40). In recent years, the watch and wait approach has been increasingly mentioned, as the survival rate among patients who received watch and wait care after initial cancer treatment is comparable to those who received radical resection (8, 9, 41). However, the risks, recovery period, and long-term impact on the function and quality of life of rectal cancer patients receiving surgery are important considerations for both surgeons and patients, especially among the elderly (41–44). Awareness of these factors may help to predict which patients are more likely to achieve pCR after nCRT, and this information may be used to counsel patients more accurately regarding treatment options.

In this study, our univariate analysis implies that mucinous adenocarcinoma, positive pre-treatment serum CEA, and higher clinical T and N stages are associated with lower odds of pCR. Our logistic regression analysis more convincingly identifies mucinous adenocarcinoma, positive pre-treatment serum CEA, and clinical T4 and N2 stages as independent clinical predictors for not achieving pCR. These results are in line with the studies mentioned above and offer additional variables that can help identify those patients who are most likely to respond to treatment, and these findings suggest which clinicopathological factors may be used for predicting pCR. Serum CEA levels are primarily used in long-term follow-up of colorectal cancer patients (14, 39), and clinicians should be alerted of the possibility of local and distant recurrence while CEA is progressively elevated. In the present study, 22% of patients with negative pre-treatment CEA levels achieved pCR, while 18% achieved pCR in the positive CEA group, and the association persisted in the regression analyses, which is consistent with the results of previous studies. This result reminds us again that CEA is not only an indicator of more advanced tumors but also an indicator of treatment response.

Mucinous adenocarcinoma of the rectum was reported to be a poor indicator for nCRT in terms of larger residual tumors, higher incidence of margin positivity, and greater residual nodal disease, but the patient population was small in reported studies (45–49). Our study included a large population number to test for the low pCR rate among mucinous adenocarcinoma patients, and the conclusion persisted in the regression analyses. Thus, in clinical practice, the watch and wait approach should be applied prudently in mucinous adenocarcinoma patients due to the low pCR rate.

### Risk Factors Among pCR Patients

Rectal cancer patients with pCR after nCRT and radical resection have better long-term outcomes than do those without pCR (4, 5). Nevertheless, there are still patients with pCR suffering from local recurrence and distant metastasis, of which the risk factors remain unrevealed. In our study, univariate and multivariate analyses using the Cox regression model imply that mucinous adenocarcinoma and clinical N2 stage might be indicative of a prognostically unfavorable biological tumor profile with a higher propensity for local or distant recurrence and decreased survival, which is consistent with the results of earlier studies (21, 25).

It has been widely reported that mucinous adenocarcinoma is a distinct pathological entity associated with poor outcome, which accounts for 5–10% of all adenocarcinomas of the rectum (50). Some researchers believe that mucinous adenocarcinomas are always associated with advanced stage at presentation and that the advanced stage of the tumor rather than its histology is responsible for the worse outcome (51, 52). The guidelines of the National Comprehensive Cancer Network (NCCN) have not ascribed mucinous histology as a risk factor that should influence therapeutic decision making. In current clinical practice, non-mucinous, and mucinous tumors are considered similar, and histology does not affect treatment decision making. However, many studies have demonstrated that mucinous histology is an independent prognostic factor (53, 54), which may have different oncogenic and molecular pathways (55). In our study, mucinous adenocarcinoma patients showed a significantly poorer outcome after nCRT and radical resection than did common adenocarcinoma patients. After Cox regression analysis, mucinous histology was still an independent risk factor, and similar reports focused on nCRT response and the prognosis of patients with mucinous adenocarcinomas are rare. Some researchers reported that mucinous tumors are always less sensitive than common adenocarcinomas are to radiation or chemotherapy (50), and mucinous tumors are more likely to relapse after radiation or chemotherapy (52). Our study also showed a worse overall outcome of mucinous rectal cancer patients after nCRT and radical resection, so the watch and wait approach should be applied more prudently in mucinous adenocarcinoma patients not only because of their low pCR rate but also because of the high relapse rate and poor overall outcome. The genetic variations of mucinous rectal cancer have been reported to affect survival, and further investigation of the relationships between genetic factors and chemoradiotherapy efficacy in mucinous adenocarcinoma is needed in the future.

### Strengths and Limitations of the Study

This study represents the largest published data set to date identify predictors of pCR and to recognize risk factors among pCR rectal cancer patients. These data were derived from the Surveillance, Epidemiology, and End Results database. SEER currently collects and publishes cancer incidence and survival data from population-based cancer registries covering ~28%of the US population.

Limitations of this study include patient selection bias and unavailable variables that may potentially be related to pCR, including information on radiation dose, chemotherapy regimens, the interval from the end of radiation to surgery, and distance from the anal verge. As the watch and wait approach for patients with a complete clinical response to nCRT has been increasingly mentioned and was attempted in some hospitals, some cCR patients may not be included in our study, causing a selection bias of patients. In our patient selection process, we found that residual tumor evaluation information in the SEER database was not available in 55% (8,000/14,555) of rectal cancer patients after nCRT, and only 45% (6,555/14,555) had available residual tumor data. The reason for residual tumor evaluation information unavailable was unknown, but it may somehow cause selection bias. With respect to the prognostic analysis of pCR patients, local and distant recurrence information was not available in the SEER database, which is also a limitation of this study. Additionally, retrospective analysis cannot offer a relationship as strong as causality. Thus, further prospective analysis is recommended for risk factor assessment.

## Conclusion

Among rectal cancer patients undergoing surgical resection after nCRT, 20.5% achieved pCR. Factors predicting a lower likelihood of pCR were mucinous adenocarcinoma, positive pre-treatment serum CEA, and clinical T4 and N2 stages. In addition, mucinous adenocarcinoma and clinical N2 stage might indicate poor prognosis. Awareness of these factors can be valuable in counseling rectal cancer patients regarding prognosis, treatment options, and follow-up plans.

## Ethics Statement

This study was approved by the ethics committee of the Second Affiliated Hospital of Zhejiang University School of Medicine.

## Author Contributions

YT and DF: designed the study. YT, DF, DL, XK, KJ, LC, YY, and KD: performed all experiments. YT, DF, DL, YY, and KD: performed the data analysis and drafted the manuscript. XK, KJ, and YY: critically revised the manuscript. All authors reviewed the manuscript.

### Conflict of Interest Statement

The authors declare that the research was conducted in the absence of any commercial or financial relationships that could be construed as a potential conflict of interest.

## Abbreviations

SEER: surveillance, epidemiology and end results
CSS: cancer-specific survival
HR: Hazard ratio
AJCC: American joint committee on cancer
CI: Confidence intervals
cCR: Clinical complete response
pCR: Pathologic complete response
nCRT: Neoadjuvant chemoradiotherapy.

## References

[1] Siegel RL Miller KD Jemal A Cancer Statistics, 2017. CA Cancer J Clinicians. (2017) 67:7–30. 10.3322/caac.21387 28055103

[2] Siegel RL Miller KD Fedewa SA Ahnen DJ Meester RGS Barzi A. Colorectal cancer statistics, 2017. CA Cancer J Clinicians. (2017) 67:177–93. 10.3322/caac.21395 28248415

[3] Ferlay J Soerjomataram I Dikshit R Eser S Mathers C Rebelo M. Cancer incidence and mortality worldwide: sources, methods and major patterns in GLOBOCAN 2012. Int J Cancer. (2015) 136:E359–86. 10.1002/ijc.29210 25220842

[4] Maas M Beets-Tan RGH Lambregts DMJ Lammering G Nelemans PJ Engelen SME. Wait-and-see policy for clinical complete responders after chemoradiation for rectal cancer. J Clin Oncol. (2011) 29:4633–40. 10.1200/JCO.2011.37.7176 22067400

[5] Maas M Nelemans PJ Valentini V Das P Rodel C Kuo LJ. Long-term outcome in patients with a pathological complete response after chemoradiation for rectal cancer: a pooled analysis of individual patient data. Lancet Oncol. (2010) 11:835–44. 10.1016/S1470-2045(10)70172-8 20692872

[6] Glynne-Jones R Wyrwicz L Tiret E Brown G Rodel C Cervantes A. Rectal cancer: ESMO clinical practice guidelines for diagnosis, treatment and follow-up. Ann Oncol. (2017) 28:iv22–40. 10.1093/annonc/mdx224 28881920

[7] Snijders HS Bakker IS Dekker JW Vermeer TA Consten EC Hoff C. High 1-year complication rate after anterior resection for rectal cancer. J Gastrointestinal Surg. (2014) 18:831–8. 10.1007/s11605-013-2381-4 24249050

[8] Glynne-Jones R Hughes R. Critical appraisal of the 'wait and see' approach in rectal cancer for clinical complete responders after chemoradiation. Br J Surg. (2012) 99:897–909. 10.1002/bjs.8732 22539154

[9] Habr-Gama A Perez RO Nadalin W Sabbaga J Ribeiro U Sousa A. Operative versus nonoperative treatment for stage 0 distal rectal cancer following chemoradiation therapy-Long-term results. Ann Surg. (2004) 240:711–7. 10.1097/01.sla.0000141194.27992.32 15383798 PMC1356472

[10] Yang TJ Goodman KA. Predicting complete response: is there a role for non-operative management of rectal cancer? J Gastroint Oncol. (2015) 6:241–6. 10.3978/j.issn.2078-6891.2014.110 25830042 PMC4311100

[11] Fu J Jiang M Tan Y Yang J Wu L Feng L. Synchronous resectable metastatic colorectal cancer: lymph node involvement predicts poor outcome. Medicine. (2015) 94:e1215. 10.1097/MD.0000000000001215 26222850 PMC4554134

[12] Fu JF Yang J Tan YN Jiang MJ Wen F Huang YQ Young patients (< = 35years old) with colorectal cancer have worse outcomes due to more advanced disease. Medicine. (2014) 93:e135 10.1097/MD.0000000000000135 25415667 PMC4616343

[13] Maggiori L Bretagnol F Aslam MI Guedj N Zappa M Ferron M. Does pathologic response of rectal cancer influence postoperative morbidity after neoadjuvant radiochemotherapy and total mesorectal excision? Surgery. (2014) 155:468–75. 10.1016/j.surg.2013.10.020 24439750

[14] Kleiman A Al-Khamis A Farsi A Kezouh A Vuong T Gordon PH. Normalization of CEA levels post-neoadjuvant therapy is a strong predictor of pathologic complete response in rectal cancer. J Gastrointest Surg. (2015) 19:1106–12. 10.1007/s11605-015-2814-3 25859755

[15] Fernandez-Acenero MJ Munoz LE Varela JS Sanchez JAC del Arco CD Paredes BG. Prognostic influence of histopathological regression patterns in rectal adenocarcinoma receiving neoadjuvant therapy. J Gastrointest Oncol. (2017) 8:49–54. 10.21037/jgo.2017.01.02 28280608 PMC5334051

[16] Yoon SM Kim DY Kim TH Jung KH Chang HJ Koom WS. Clinical parameters predicting pathologic tumor response after preoperative chemoradiotherapy for rectal cancer. Int J Radiat Oncol Biol Phys. (2007) 69:1167–72. 10.1016/j.ijrobp.2007.04.047 17967307

[17] Garland ML Vather R Bunkley N Pearse M Bissett IP. Clinical tumour size and nodal status predict pathologic complete response following neoadjuvant chemoradiotherapy for rectal cancer. Int J Colorectal Dis. (2014) 29:301–7. 10.1007/s00384-013-1821-7 24420737

[18] Wallin U Rothenberger D Lowry A Luepker R Mellgren A. CEA-a predictor for pathologic complete response after neoadjuvant therapy for rectal cancer. Dis Colon Rectum. (2013) 56:859–68. 10.1097/DCR.0b013e31828e5a72 23739192

[19] Huh JW Kim HR Kim YJ. Clinical prediction of pathological complete response after preoperative chemoradiotherapy for rectal cancer. Dis Colon Rectum. (2013) 56:698–703. 10.1097/DCR.0b013e3182837e5b 23652742

[20] Shahab D Gabriel E Attwood K Ma WW Francescutti V Nurkin S. Adjuvant chemotherapy is associated with improved overall survival in locally advanced rectal cancer after achievement of a pathologic complete response to chemoradiation. Clin Colorectal Cancer. (2017) 16:300–7. 10.1016/j.clcc.2017.03.005 28420585

[21] Zorcolo L Rosman AS Restivo A Pisano M Nigri GR Fancellu A. Complete pathologic response after combined modality treatment for rectal cancer and long-term survival: a meta-analysis. Ann Surg Oncol. (2012) 19:2822–32. 10.1245/s10434-011-2209-y 22434243

[22] Wolthuis AM Penninckx F Haustermans K De Hertogh G Fieuws S Van Cutsem E. Impact of interval between neoadjuvant chemoradiotherapy and TME for locally advanced rectal cancer on pathologic response and oncologic outcome. Ann Surg Oncol. (2012) 19:2833–41. 10.1245/s10434-012-2327-1 22451236

[23] Tulchinsky H Shmueli E Figer A Klausner JM Rabau M. An interval > 7 weeks between neoadjuvant therapy and surgery improves pathologic complete response and disease-free survival in patients with locally advanced rectal cancer. Ann Surg Oncol. (2008) 15:2661–7. 10.1245/s10434-008-9892-3 18389322

[24] Pucciarelli S Capirci C Emanuele U Toppan P Friso ML Pennelli GM. Relationship between pathologic T-stage and nodal metastasis after preoperative chemoradiotherapy for locally advanced rectal cancer. Ann Surg Oncol. (2005) 12:111–6. 10.1245/ASO.2005.03.044 15827790

[25] Lorimer PD Motz BM Kirks RC Boselli DM Walsh KK Prabhu RS. Pathologic complete response rates after neoadjuvant treatment in rectal cancer: an analysis of the national cancer database. Ann Surg Oncol. (2017) 24:2095–103. 10.1245/s10434-017-5873-8 28534080

[26] Kim HJ Choi GS Park JS Park S Kawai K Watanabe T. Clinical significance of thrombocytosis before preoperative chemoradiotherapy in rectal cancer: predicting pathologic tumor response and oncologic outcome. Ann Surg Oncol. (2015) 22:513–9. 10.1245/s10434-014-3988-8 25145505

[27] Al-Sukhni E Attwood K Mattson DM Gabriel E Nurkin SJ. Predictors of pathologic complete response following neoadjuvant chemoradiotherapy for rectal cancer. Ann Surg Oncol. (2016) 23:1177–86. 10.1245/s10434-015-5017-y 26668083 PMC5295136

[28] Yeo SG Kim DY Kim TH Chang HJ Oh JH Park W. Pathologic complete response of primary tumor following preoperative chemoradiotherapy for locally advanced rectal cancer long-term outcomes and prognostic significance of pathologic nodal status (KROG 09-01). Ann Surg. (2010) 252:998–1004. 10.1097/SLA.0b013e3181f3f1b1 21107110

[29] Kalady MF de Campos-Lobato LF Stocchi L Geisler DP Dietz D Lavery IC. Predictive factors of pathologic complete response after neoadjuvant chemoradiation for rectal cancer. Ann Surg. (2009) 250:582–9. 10.1097/SLA.0b013e3181b91e63 19710605

[30] Lee KH Kim JC Kim JY Kim JS. Oncologic results and prognostic predictors of patients with locally advanced rectal cancer showing ypN0 after radical surgery following neoadjuvant chemoradiotherapy. Int J Colorectal Dis. (2015) 30:1041–50. 10.1007/s00384-015-2261-3 26002751

[31] Monson JRT Probst CP Wexner SD Remzi FH Fleshman JW Garcia-Aguilar J. Failure of evidence-based cancer care in the united states the association between rectal cancer treatment, cancer center volume, and geography. Ann Surg. (2014) 260:625–32. 10.1097/SLA.0000000000000928 25203879

[32] Park YA Sohn SK Seong J Baik SH Lee KY Kim NK. Serum CEA as a predictor for the response to preoperative chemoradiation in rectal cancer. J Surg Oncol. (2006) 93:145–50. 10.1002/jso.20320 16425302

[33] Petrelli F Sgroi G Sarti E Barni S. Increasing the interval between neoadjuvant chemoradiotherapy and surgery in rectal cancer a meta-analysis of published studies. Ann Surg. (2016) 263:458–64. 10.1097/SLA.0000000000000368 24263329

[34] Probst CP Becerra AZ Aquina CT Tejani MA Wexner SD Garcia-Aguilar J. Extended intervals after neoadjuvant therapy in locally advanced rectal cancer: the key to improved tumor response and potential organ preservation. J Am College Surg. (2015) 221:430–40. 10.1016/j.jamcollsurg.2015.04.010 26206642 PMC5014360

[35] Robbins AS Pavluck AL Fedewa SA Chen AY Ward EM. Insurance status, comorbidity level, and survival among colorectal cancer patients age 18 to 64 years in the national cancer data base from 2003 to 2005. J Clin Oncol. (2009) 27:3627–33. 10.1200/JCO.2008.20.8025 19470927

[36] Sloothaak DAM Geijsen DE van Leersum NJ Punt CJA Buskens CJ Bemelman WA. Optimal time interval between neoadjuvant chemoradiotherapy and surgery for rectal cancer. Br J Surg. (2013) 100:933-U108. 10.1002/bjs.9112 23536485

[37] Smith JD Ruby JA Goodman KA Saltz LB Guillem JG Weiser MR. Nonoperative management of rectal cancer with complete clinical response after neoadjuvant therapy. Ann Surg. (2012) 256:965–72. 10.1097/SLA.0b013e3182759f1c 23154394

[38] Stipa F Zernecke A Moore HG Minsky BD Wong WD Weiser M. Residual mesorectal lymph node involvement following neoadjuvant combined-modality therapy: rationale for radical resection? Ann Surg Oncol. (2004) 11:187–91. 10.1245/ASO.2004.06.010 14761922

[39] Yang KL Yang SH Liang WY Kuo YJ Lin JK Lin TC. Carcinoembryonic antigen (CEA) level, CEA ratio, and treatment outcome of rectal cancer patients receiving pre-operative chemoradiation and surgery. Radiat Oncol. (2013) 8:43. 10.1186/1748-717X-8-43 23452434 PMC3599903

[40] Zeng WG Zhou ZX Liang JW Wang Z Hou HR Zhou HT. Impact of interval between neoadjuvant chemoradiotherapy and surgery for rectal cancer on surgical and oncologic outcome. J Surg Oncol. (2014) 110:463–67. 10.1002/jso.23665 24889826

[41] Sabbaga J Braghiroli MI Hoff PM. Is surgery always necessary in rectal cancer? Oncology. (2014) 28:607–11. 10.1016/j.cellsig.2005.10.018 25144282

[42] Ackerman SJ Daniel S Baik R Liu E Mehendale S Tackett S. Comparison of complication and conversion rates between robotic-assisted and laparoscopic rectal resection for rectal cancer: which patients and providers could benefit most from robotic-assisted surgery? J Med Econ. (2018) 21:254–61. 10.1080/13696998.2017.1396994 29065737

[43] Kilic D Yalman D Aksu G Atasoy BM Igdem S Dincbas FO. Impact of adjuvant chemoradiotherapy for rectal cancer on the long-term quality of life and late side effects: a multicentric clinical evaluation by the Turkish oncology group. Asian Pac J Cancer Prev. (2012) 13:5741–6. 10.7314/APJCP.2012.13.11.5741 23317249

[44] Bakker IS Snijders HS Wouters MW Havenga K Tollenaar RA Wiggers T. High complication rate after low anterior resection for mid and high rectal cancer; results of a population-based study. Eur J Surg Oncol. (2014) 40:692–8. 10.1016/j.ejso.2014.02.234 24655803

[45] Ling CR Wang R Wang MJ Ping J Zhuang W. Prognosis and value of preoperative radiotherapy in locally advanced rectal signet-ring cell carcinoma. Sci Rep. (2017) 7:45334. 10.1038/srep45334 28345614 PMC5366911

[46] Oberholzer K Menig M Pohlmann A Junginger T Heintz A Kreft A. Rectal cancer: assessment of response to neoadjuvant chemoradiation by dynamic contrast-enhanced MRI. J Magnet Reson Imag. (2013) 38:119–26. 10.1002/jmri.23952 23188618

[47] Oberholzer K Menig M Kreft A Schneider A Junginger T Heintz A. Rectal cancer: mucinous carcinoma on magnetic resonance imaging indicates poor response to neoadjuvant chemoradiation. Int J Radiat Oncol Biol Phys. (2012) 82:842–8. 10.1016/j.ijrobp.2010.08.057 21236593

[48] Newton AD Li J Jeganathan AN Mahmoud NN Epstein AJ Paulson EC. A Nomogram to predict lymph node positivity following neoadjuvant chemoradiation in locally advanced rectal cancer. Dis Colon Rectum. (2016) 59:710–7. 10.1097/DCR.0000000000000638 27384088

[49] Bitterman DS Resende Salgado L Moore HG Sanfilippo NJ Gu P Hatzaras I. Predictors of complete response and disease recurrence following chemoradiation for rectal cancer. Front Oncol. (2015) 5:286. 10.3389/fonc.2015.00286 26734570 PMC4686647

[50] Simha V Kapoor R Gupta R Bahl A Nada R. Mucinous adenocarcinoma of the rectum: a poor candidate for neo-adjuvant chemoradiation? J Gastroint Oncol. (2014) 5:276–9. 10.3978/j.issn.2078-6891.2014.020 25083301 PMC4110493

[51] Nitsche U Zimmermann A Spath C Muller T Maak M Schuster T. Mucinous and signet-ring cell colorectal cancers differ from classical adenocarcinomas in tumor biology and prognosis. Ann Surg. (2013) 258:775–82; discussion 782–773. 10.1097/SLA.0b013e3182a69f7e 23989057 PMC3888475

[52] Lee DW Han SW Lee HJ Rhee YY Bae JM Cho NY. Prognostic implication of mucinous histology in colorectal cancer patients treated with adjuvant FOLFOX chemotherapy. Br J Cancer. (2013) 108:1978–84. 10.1038/bjc.2013.232 23652310 PMC3670503

[53] Verhulst J Ferdinande L Demetter P Ceelen W. Mucinous subtype as prognostic factor in colorectal cancer: a systematic review and meta-analysis. J Clin Pathol. (2012) 65:381–8. 10.1136/jclinpath-2011-200340 22259177

[54] Mekenkamp LJ Heesterbeek KJ Koopman M Tol J Teerenstra S Venderbosch S. Mucinous adenocarcinomas: poor prognosis in metastatic colorectal cancer. Eur J Cancer. (2012) 48:501–9. 10.1016/j.ejca.2011.12.004 22226571

[55] Kim SH Shin SJ Lee KY Kim H Kim TI Kang DR. Prognostic value of mucinous histology depends on microsatellite instability status in patients with stage III colon cancer treated with adjuvant FOLFOX chemotherapy: a retrospective cohort study. Ann Surg Oncol. (2013) 20:3407–13. 10.1245/s10434-013-3169-1 23943026

[56] Tan YN Fu DL Jiang K Kong XX Yuan Y Ding KF Predictors and risk factors of pathologic complete response following neoadjuvant chemoradiotherapy for rectal cancer. Ann Oncol. (2018) 29:1 10.1093/annonc/mdy374.059 PMC6585388 31263674

