# Supplementary material for: Predictors and Risk Factors of Pathologic Complete Response Following Neoadjuvant Chemoradiotherapy for Rectal Cancer: A Population-Based Analysis
Source: Front Oncol. 2019 Jun 13;9:497. doi: 10.3389/fonc.2019.00497 (PMC6585388; doi:10.3389/fonc.2019.00497)

Supplement table 1. Clinical and pathological characteristics of pCR patients

| Variable | Common  adenocarcinoma(%) | Mucinous  adenocarcinoma(%) | *P* value |
| --- | --- | --- | --- |
| Age |  |  | 0.841 |
| <=50yrs | 275(21%) | 12(20%) |  |
| >50yrs | 1008(79%) | 47(80%) |  |
| Gender |  |  | 0.814 |
| Male | 498(39%) | 22(37%) |  |
| Female | 785(61%) | 37(63%) |  |
| Marital status |  |  | 0.412 |
| Married | 773(60%) | 31(53%) |  |
| Single | 198(15%) | 8(14%) |  |
| Divorced | 242(19%) | 16(27%) |  |
| Unknown | 70(5%) | 4(7%) |  |
| Race |  |  | 0.804 |
| White | 1038(81%) | 49(83%) |  |
| Black | 90(7%) | 5(8%) |  |
| Other | 148(12%) | 5(8%) |  |
| Unknown | 7(1%) | 0(0%) |  |
| Differentiation | |  | <0.001 |
| Well | 82(6%) | 1(2%) |  |
| Moderate | 877(68%) | 31(53%) |  |
| Poor | 106(8%) | 15(25%) |  |
| Undifferentiated | 8(1%) | 3(5%) |  |
| Unknown | 210(16%) | 9(15%) |  |
| CEA |  |  | 0.650 |
| Negative | 522(41%) | 21(36%) |  |
| Positive | 330(26%) | 18(31%) |  |
| Unknown | 431(34%) | 20(34%) |  |
| cTstage |  |  | 0.042 |
| 1 | 104(8%) | 0(0%) |  |
| 2 | 186(14%) | 7(12%) |  |
| 3 | 929(72%) | 46(78%) |  |
| 4 | 64(5%) | 6(10%) |  |
| cNstage |  |  | 0.013 |
| 0 | 703(55%) | 26(44%) |  |
| 1 | 490(38%) | 23(39%) |  |
| 2 | 90(7%) | 10(17%) |  |
| Total | 1283(100%) | 59(100%) |  |

Supplement table 2. Point assignment and score in the nomogram

| Factors | score |
| --- | --- |
| Histology |  |
| Common adenocarcinoma | 30 |
| Mucinous adenocarcinoma | 0 |
| Differentiation |  |
| Well | 20 |
| Moderate | 17 |
| Poor | 22 |
| Undifferentiated | 0 |
| Unknow | 50 |
| CEA |  |
| Negative | 13 |
| Positive | 0 |
| Unknow | 9 |
| cTstage |  |
| T0 | 100 |
| T1 | 55 |
| T2 | 47 |
| T3 | 39 |
| T4 | 0 |
| cNstage |  |
| N0 | 19 |
| N1 | 27 |
| N2 | 0 |
| Unknow | 6 |
| Total point | Probability of achieving pCR |
| 9 | 0.05 |
| 59 | 0.1 |
| 113 | 0.2 |
| 149 | 0.3 |
| 179 | 0.4 |
| 206 | 0.5 |
| 233 | 0.6 |

Supplement figure 1: The calibration curve for predicting probability of achieving pCR in the primary cohort.


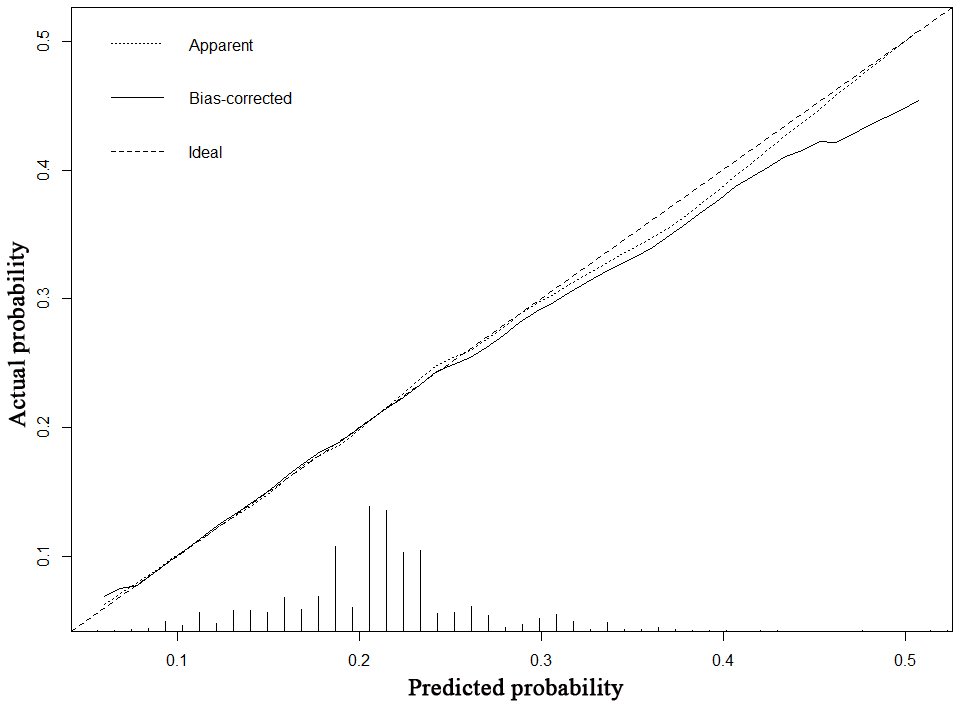

Supplement: Supplementary file 1 [file Data_Sheet_1.docx]
